# Lignocellulosic Biomass of C3 and C4 Perennial Grasses as a Valuable Feedstock for Particleboard Manufacture

**DOI:** 10.3390/ma15186384

**Published:** 2022-09-14

**Authors:** Dominika Janiszewska, Grzegorz Żurek, Danuta Martyniak, Wojciech Bałęczny

**Affiliations:** 1 Łukasiewicz Research Network, Poznań Institute of Technology, Center of Wood Technology, Winiarska St.1, 60-654 Poznan, Poland; 2 Plant Breeding and Acclimatization Institute, National Research Institute, Radzików, 05-870 Błonie, Poland

**Keywords:** switchgrass, miscanthus, tall wheatgrass, lignocellulosic biomass, wood-based composites, formaldehyde

## Abstract

Looking for new alternative raw materials is one of the key issues in line with a bioeconomy approach, particularly for particleboard manufacturing. In this framework, this paper presents a comparison of some physico-mechanical properties and the formaldehyde contents of particleboards made with 30% substitution of grass biomass from six perennial grass species. Our studies indicate relatively high values of mechanical properties for particleboards made with the addition of biomass from grasses with the C_4_ photosynthetic pathway: *Miscanthus x giganteus* and switchgrass (*Panicum virgatum*). Boards made with the addition of biomass from grasses with the C_3_ photosynthetic pathway—tall wheatgrass (*Elymus elongatus*), tall fescue (*Festuca arundinacea*), and perennial ryegrass (*Lolium perenne*)—gave lower values of mechanical properties. The opposite results were obtained in the case of the formaldehyde content: the lowest value was measured for particleboards made with the addition of tall fescue biomass (0.1% less than the control), and the highest for switchgrass (0.9% greater than the control) and cordgrass (3.2% greater than the control). Future research should address the optimization of the manufacturing process of particleboards from perennial grasses, taking into account the needs and technical possibilities of the wood industry sector.

## 1. Introduction

Particleboards are panels produced by combining wooden particles with a proper glue and they are among the most important value-added products in the wood sector [1,2].

On the one hand, global consumption of wood is expected to increase in the near future [3,4,5,6], but on the other hand, it is also important to safeguard forests, in order to allow them to play their crucial role in the context of climate change mitigation by storing atmospheric CO_2_ [7,8,9].

To avoid conflict between environmental requirements and the production of wood for energy, paper, furniture and construction materials, new biomass sources have to be found, evaluated, and converted to value-added products [10,11,12,13,14]. Moreover, the timber market is subject to continuously increasing demand from both the construction and energy industries, leading to a shortage of wood particles and higher prices [15,16].

Therefore, particleboard producers need to look for new, cheap, and available sources of raw material in order to ensure the sustainability of the manufacturing process without compromising the quality of the obtained boards [2,17,18,19].

Apart from wood there are many possible feedstocks for producing particleboards, including post-consumer wood, forest, and agricultural residues [1,20].

The first attempts to produce particleboards from cereal straw revealed the low suitability of such feedstock, due to its large capillaries, which cause the collection of an excessive amount of resin, and to poor adhesion, an effect of the high content of mineral substances [21]. The low mechanical quality of panels containing wheat (*Triticum* spp.) and rapeseed (*Brassica napus* L.) straw was confirmed more recently, with internal bond strength values in the low range of 61–99 mN mm^−2^, notwithstanding the application of enzymes to increase particle surface energy [22].

Other lignocellulose raw materials utilized for particleboard manufacture include bagasse and flax shives [17,23,24]. In particular, Pozzer et al. reported bending properties similar to those of commercial particleboards for trapezoidal core sandwich panels based on sugarcane (*Saccharum officinarum* L.) bagasse agglomerated with castor (*Ricinus communis* L.) oil-based polyurethane resin [24]. A study by a group of Japanese and Indonesian scientists showed that particleboards produced from the inner part of oil palm (*Elaeis guineensis* L.) trunk attained acceptable physical and mechanical properties, even if this required the addition of ammonium dihydrogen phosphate [25].

Among the different sources of biomass, perennial grasses are used increasingly worldwide, and many of them can be grown with minimal maintenance on marginal soils [26], which is a fundamental criterion for the sustainability of industrial crop cultivation [27].

In this study, among many grass species, focus was placed on certain species with relatively high biomass potential in different soil quality conditions: miscanthus (*Miscanthus x giganteus* Greef & Deuter), switchgrass (*Panicum virgatum* L.) and prairie cordgrass (*Spartina pectinata* Bosc ex Link), these being warm-season grasses with the C_4_ photosynthetic pathway (hereafter referred to as C_4_ grasses); and tall wheatgrass (*Elymus elongatus* (Host.) Runemark), tall fescue (*Festuca arundinacea* Schreb.) and perennial ryegrass (*Lolium perenne* L.), these being cool-season grasses with the C_3_ photosynthetic pathway (hereafter referred to as C_3_ grasses).

Perennial grass biomass has been successfully tested for different applications, including as a supplement to wood in the production of medium-density fiberboard (MDF) [28], fiber-reinforced composites [29], and insulation and composite boards [30,31]. However, few studies have been carried out to evaluate the suitability of perennial grass biomass for particleboard production.

One such species which has been briefly evaluated in that regard is miscanthus [32,33,34,35]. Miscanthus has been investigated in Europe as a potential source of fiber for composite materials such as MDF and chipboard, pulp for paper and packaging, biodegradable geotextiles (e.g., for temporary protection of slopes and banks), filters and sorbents, and insulation [32,34,35,36,37]. Moderate mechanical performance has been reported, mostly due to the low density [34,35].

Switchgrass may be a fiber source requiring fewer inputs for growth and may be economically more viable than traditional fiber crops such as jute and flax. In addition, about 25–30% of switchgrass can be obtained as long fibers for high-value applications, and another 20–25% consisting of short fibers and hemicellulose can still be used for ethanol production [38]. A single plant producing two types of fibers with such distinct characteristics is a unique phenomenon [38]. Low growing costs, high fiber yield (20–25%), and distinct fiber properties make switchgrass a crop with high potential for use in construction materials and fiber production.

Tall wheatgrass biomass can be used to produce certain kinds of paper, cardboard products, MDF, and particleboards [39]. It has also been tested for renewable energy production and paper-making [40,41].

Perennial ryegrass clippings have been incorporated into particleboards together with eucalyptus [42]. Boards manufactured with 100% grass clippings exhibited the lowest quality. The overall panel properties improved when a lower percentage of grass clippings was added. Based on initial results, it appears that grass should make up no more than 13% to achieve acceptable panel properties for interior fitments and general uses.

No sufficient information is available on the evaluation of cordgrass and tall fescue biomass for particleboard production.

Taking the above into account, there is a need to improve existing knowledge on the possibility of using perennial grass biomass for particleboard production. An additional argument supporting the intensification of research on the use of grass biomass in the particleboards production is the relatively low (compared to other types of agricultural crops) impact of these crops on the natural environment. It results from their perennially, low expenditure on fertilization and chemicals, and most importantly, the possibility of growing on marginal lands unsuitable for food production.

The objective of this study was to investigate some physical and mechanical properties, as well as formaldehyde contents of particleboards made using biomass from six perennial grass species with different photosynthetic pathways.

The novelty of our work lies in the comparison of two groups of grass species defined on the basis of their photosynthetic pathways, which may be one of the main factors determining a plant’s development, and therefore its final applications. Moreover, grass species as *Spartina pectinata*, *Festuca arundinacea*, and *Lolium perenne* have not so far been widely tested for their suitability for particleboard manufacture.

## 2. Materials and Methods

### 2.1. Plant Establishment and Raw Material Collection

Seed accessions of tall wheatgrass cv BAMAR, perennial ryegrass cv BARONKA, and tall fescue cv BAROLEX were kindly provided by breeders (Table 1). Seed of switchgrass cv MARDAN originated from the Department of Grasses, Legumes, and Energy Plants of PB&AI, NRI.

To produce new planting material of miscanthus and cordgrass, three-year-old plants were split whilst dormant (late April 2014) and the rhizome pieces were collected for re-planting. At the end of February 2014, seeds of switchgrass cv MARDAN were sown on a germination tank, and after a few days, emerging seedlings were transplanted to plastic pots filled with a sand–soil–peat mixture. After 3 months of vegetation in an unheated glasshouse, plants were transferred to a field. Seed, seedlings, and rhizomes of all grass species selected for our experiment were placed in a field in Radzików, Central Poland (52°12′44″ N, 20°38′14″ E) in sandy clay soil (alfisol) with the following parameters: pH_KCl_—5.17; C_org_—0.35%; clay—12.7%, silt—40.0%, sand—47.3%; macronutrients (in mg·kg^−1^ of soil): NH_4_—8.0, P—63.0, K—80.0, Ca—330.0, Mg—58.5, Cl—14.5. Establishment details are given in Table 1. For each species, a 100 m^2^ plot was established.

The plants were grown for the next three years to determine their yield potential. Weeds were hand-removed, and mineral fertilizers were applied in spring each year in the following amounts (in kg·ha^−1^): 40 N, 60 P, and 60 K. No additional chemicals (herbicides or pesticides) were used. Each year, biomass was mechanically cut and removed: in autumn at seed maturity for the C_3_ species and at the end of winter for the C_4_ species. For particleboard testing, the biomass of C_3_ species was collected in 2016, and that of C_4_ species in 2017. Each plot was divided into three sub-plots. From each sub-plot, ca. 10 m^2^ was selected for biomass cutting for use in the experiment. The biomass collected from the sub-plots was further mixed together to produce a representative sample for each species. For each species, ca. 20 kg of air-dried biomass was used for particleboard testing. Biomass was harvested using an ALKO 5001 R-II reel lawn mower (ALKO, Sachsen, Germany) and then dried to a humidity of 10% and chopped into pieces of 1.5 to 2.0 cm using an MTD 475 petrol-powered shredder (Briggs and Stratton, Viernheim, Germany) dedicated for the disintegration of tree branches.

### 2.2. Particleboard Production

Particleboards were produced using industrial wood particles from a leading Polish manufacturer of particleboards, and particles of alternative lignocellulosic raw material shredded in a laboratory knife ring flaker. The particles obtained from the lignocellulosic raw material were passed through sieves with a mesh size of 0.5 mm, and the fractions remaining on the sieve were used in the production of panels. Wood particles and alternative raw materials were dried at 100 °C to a moisture content of 2%. Single-layer particleboards with an average density of 670 kg·m^−3^ and dimensions 700 mm × 500 mm × 16 mm were produced under laboratory conditions. Detailed parameters of particleboard production are given in Table 2.

Melamine–urea formaldehyde (MUF) adhesive was used in the production of the particleboards; its properties are given in Table 3.

### 2.3. Experimental Design and Data Analysis

Wood particles from a manufacturer of particleboards and six alternative raw materials were used in the research. With each alternative raw material, a slab was produced in which the grass particles accounted for 30% by weight of the lignocellulosic raw material. For purposes of comparison, a reference board was produced, which was made only of wood particles. The compositions of the boards produced are given in Table 4.

### 2.4. Determination of Standard Properties of Particleboards

Standard chemical, physical, and mechanical properties of the manufactured boards were evaluated, including:

- Modulus of rupture (MOR) and modulus of elasticity (MOE) according to EN 310:1994,
- Internal bond strength (IB) according to EN 319:1999,
- Thickness swelling (TS) and water absorption (WA) after 24 h based on EN 317:1999,
- Formaldehyde content (FC) according to EN ISO 12460-5:2016-02, and
- Density according to EN 323:1999.

#### 2.4.1. Mechanical Properties

Three-point bending tests (EN 310) and internal bond strength (IB) tests were performed on a Zwick testing machine using testXpert software (Genova, Italy). Samples with dimensions (L × W × T) 370 mm × 50 mm × 16 mm were used to determine modulus of rupture (MOR) and modulus of elasticity (MOE), being subjected to a loading rate of 7 mm·min^−1^ until failure. Square samples with a side length of 50 mm were used to determine the internal bond strength (IB). The samples were sanded and glued between stainless steel blocks. Blocks were positioned in holders and preloaded at a tension of 5 N, and then a loading rate of 1 mm·min^−1^ was applied until failure.

The following standards were used: EN 312 Particleboards and fiberboards—determination of swelling in thickness after immersion in water, 1993; EN 310 Wood-based panels—determination of modulus of elasticity in bending and bending strength; EN 319 Particleboards and fiberboards—determination of tensile strength perpendicular to the plane of the board, 1993; and EN 312 Particleboards—Specifications.

#### 2.4.2. Water Absorption and Thickness Swelling

Thickness swelling (TS) and water absorption (WA) were determined based on EN 317. Square samples with a side length of 50 mm and thickness 16 mm were fully immersed in water at 20 °C. After an immersion time of 24 h, the samples were taken out of the water and excess water was removed. TS was measured using a thickness gauge, positioned in the center of the samples (with a precision of 0.01 mm). WA was determined by weighing the samples using laboratory scales before and after immersion (with a precision of 0.001 g).

#### 2.4.3. Density of Finished Particleboards

Three square samples with side length 50 mm and thickness 16 mm were used to determine density profiles. An X-ray scanning device (GreCon, Alfeld/Hannover, Germany) was used to determine the density profile. Density measurements were made every 0.02 mm at a measurement speed of 0.4 mm·s^−1^.

#### 2.4.4. Formaldehyde Content

Formaldehyde content was determined by means of extraction in a perforator, according to EN 12460-5. Approximately 110 g of 25 mm × 25 mm specimens were placed for 2 h in boiling toluene (600 mL). The refluxing toluene was bubbled through distilled water to extract any dissolved formaldehyde. The formaldehyde content of aqueous solution was determined photometrically and was expressed as milligrams of free formaldehyde per 100 g of dry board.

### 2.5. Statistical Analysis

All calculations were performed using STATISTICA 12 ^®^ for Windows (StatSoft, Inc., 2300 East 14th St. Tulsa, OK 74104, USA). Differences were regarded as significant at a level of 95% probability. Least significant differences (LSD) were calculated according to the Tukey test.

## 3. Results and Discussion

### 3.1. Yield Potential

**Figure 1:**
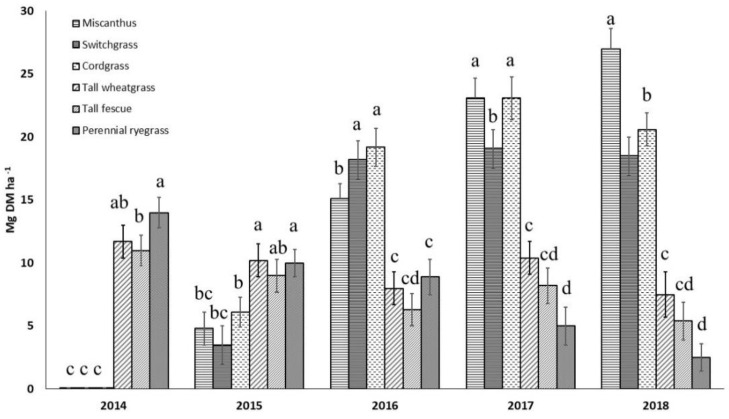
Biomass yields (in Mg DM ha^−1^) of six perennial grasses from the year of establishment of plots. Vertical bars indicate standard deviations. Different letters indicate the presence of statistically significant differences at *p* < 0.05 according to one-way ANOVA and Tukey test.

Biomass yields of C4 grass species increased from the second year after establishment and reached their peak after 4 years (Figure 1). The estimated duration of C4 grass species plantations is from 10 up to 20 years, depending on species and growing conditions (climate, soil, management) [43,44]. The C4 species gave no yields in the year of establishment, as they were harvested at the end of winter in the following year. However, C3 grass species gave high biomass yields in the year of establishment, with decreasing yields after 2–3 years of cultivation. The generally high yields obtained from all the investigated species further confirmed the suitability of perennial grasses to provide high amount of biomass in a short-time span in the context of Central European Agriculture [45,46,47].

### 3.2. Properties of Particleboards

The statistical analysis showed that in the case of MOR, MOE, and IB there were significant differences between particleboards made with the addition of C_4_ and C_3_ grass biomass. In the case of the aforementioned traits as well as TS and WA, significant differences were also identified between grass species within the C_3_ and C_4_ groups.

**Figure 2:**
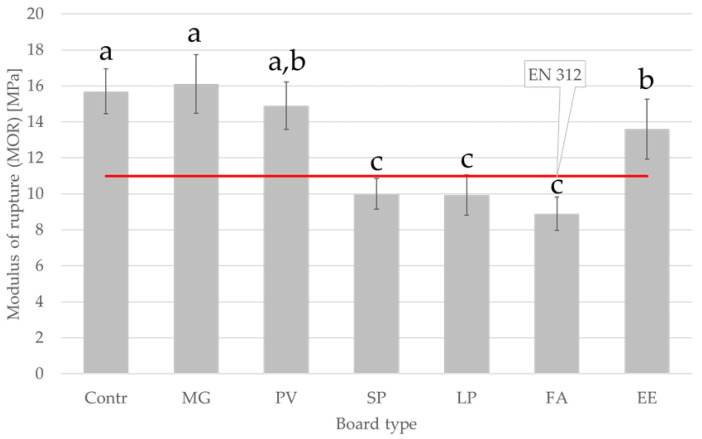
Modulus of rupture of manufactured panels. Abbreviations used: contr.—control (100% wood); FA—*Festuca arundinacea*, EE—*Elymus elongatus*, LP—*Lolium perenne*, MG—*Miscanthus x giganteus*, PV—*Panicum virgatum*, SP—*Spartina pectinate.* Different letters indicate the presence of statistically significant differences at *p* < 0.05 according to one-way ANOVA and Tukey test.

**Figure 3:**
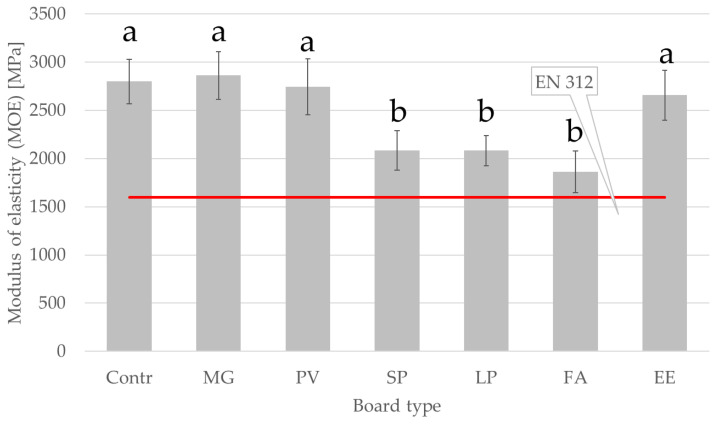
Modulus of elasticity of manufactured panels. Abbreviations used: contr.—control (100% wood); FA—*Festuca arundinacea*, EE—*Elymus elongatus*, LP—*Lolium perenne*, MG—*Miscanthus x giganteus*, PV—*Panicum virgatum*, SP—*Spartina pectinate.* Different letters indicate the presence of statistically significant differences at *p* < 0.05 according to one-way ANOVA and Tukey test.

Figure 2 and Figure 3 show MOR and MOE values for the manufactured panels. The highest values of MOR (16.1 MPa) and MOE (2861 MPa) were determined for MG board, which contained 30% miscanthus particles, resulting much higher than the values reported for pure miscanthus particleboards, i.e., about 14 MPa for MOR and about 1600 MPa for MOE [35]. On the other hand, obtained data for switchgrass particleboards are consistent with current literature [39]. The lowest values of MOR (8.9 MPa) and MOE (1863 MPa) were obtained for FA particleboard, which was produced using industrial wood particles with a 30% addition of tall fescue particles. The average MOR value for C_4_ grass species (13.7 MPa) differed significantly from the value obtained for C_3_ grass species (10.9 MPa). The results indicate that the addition of selected alternative lignocellulosic raw materials to particleboards may reduce the MOR and MOE values, except in the case of the board with the addition of miscanthus particles. The SP, LP, and FA boards do not meet the minimum bending strength requirements for P2 boards (boards for interior fitments, including furniture) according to EN 312.

Based on the EN 312 standard, the minimum MOR and MOE values of particleboards for interior fitments including furniture are 11 MPa and 1600 MPa, respectively. For the SP, LP, and FA boards, these parameters are lower than for the other boards but the obtained values are above the minimum requirements.

The lower MOR and MOE values may be due to the smaller specific surface area and less slender particles of the alternative raw materials added to the particleboards. Consequently, the SP, LP, and FA boards exhibited lower strength after the addition of 30% particles from alternative raw materials. The particle geometry used in particleboard production has a much greater influence on the board properties than the mechanical properties of the particles. The strength and stiffness of particleboards are affected by the quality of the bonds between the particles, which in turn are significantly influenced by their dimensions. In addition to differences in the size and shape of particles, the parameters of the board are also affected by their moisture content [48]. The size of the obtained particles is influenced by the specific weight of the raw material—during the grinding process (flaking), the specific weight and hardness of the raw material determine the ability of the blade to penetrate the lignocellulosic material. Other parameters that may affect the size of the obtained particles include the moisture content of the raw material, knife angle, feeding and cutting speed, and temperature conditions [49].

**Figure 4:**
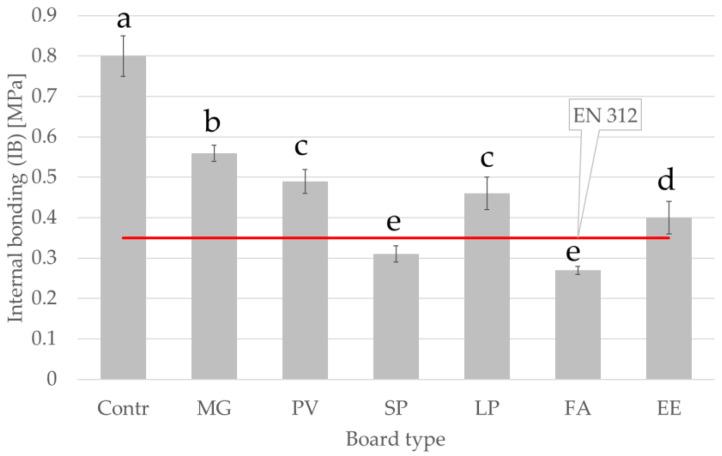
Internal bond strength of manufactured panels. Abbreviations used: contr.—control (100% wood); FA—*Festuca arundinacea*, EE—*Elymus elongatus*, LP—*Lolium perenne*, MG—*Miscanthus x giganteus*, PV—*Panicum virgatum*, SP—*Spartina pectinate.* Different letters indicate the presence of statistically significant differences at *p* < 0.05 according to one-way ANOVA and Tukey test.

The obtained internal bond strength (IB) values are shown in Figure 4. Significant differences in the IB values of boards with the addition of grasses were identified within the C_3_ and C_4_ groups. Significant differences were also observed between the mean IB values of the C_3_ and C_4_ boards. The highest IB value for particleboards with added alternative particles was observed for MG board (0.56 MPa) and the lowest for FA board (0.28 MPa). The addition of 30% alternative particles reduced the IB value compared with the control board. The SP and FA boards did not reach the minimum internal bond strength value for boards for interior fitments including furniture, which should be at least 0.35 MPa according to EN 312.

The lower IB values for boards with the addition of alternative raw materials may be due to the larger particle surface area per unit mass compared with wood particles, which means that less adhesive is deposited on the surfaces of the particles from alternative raw materials [50]. It is predicted that increasing the resination ratio will increase the IB value.

In the current literature, different studies presented trials to increase the mechanical properties of particleboards from alternative biomasses [51], for instance by surface layer treatment [52], carbonization treatment [53] or, when dealing with agro-wastes such as corn stalks, biomass fermentation [54].

Comparing the obtained data with current literature, it is possible to notice that the presented value of IB for miscanthus-based particleboards were almost 45% higher than the values found by Klímek et al. (2018) [35]. However, there is the need to highlight that the study from Klímek et al. (2018) applied different manufacturing parameters, methylene diphenyl diioscyanate as adhesive, and the panels were based on pure miscanthus particles.

**Figure 5:**
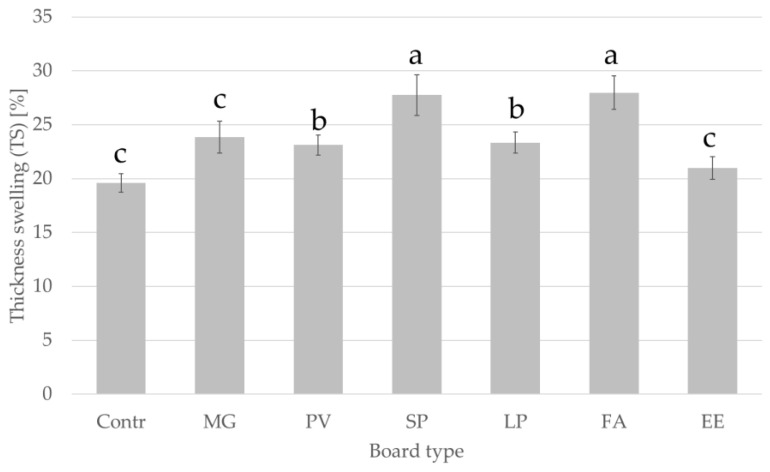
Thickness swelling of manufactured panels after 24 h. Abbreviations used: contr.—control (100% wood); FA—*Festuca arundinacea*, EE—*Elymus elongatus*, LP—*Lolium perenne*, MG—*Miscanthus x giganteus*, PV—*Panicum virgatum*, SP—*Spartina pectinate.* Different letters indicate the presence of statistically significant differences at *p* < 0.05 according to one-way ANOVA and Tukey test.

**Figure 6:**
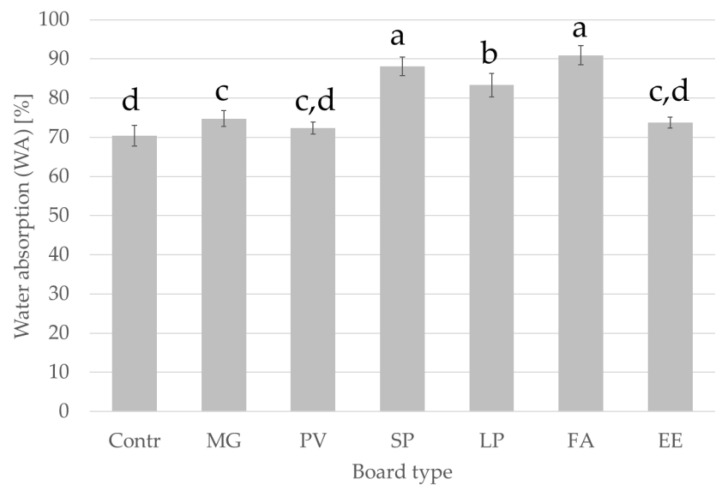
Water absorption of manufactured panels after 24 h. Abbreviations used: contr.—control (100% wood); FA—*Festuca arundinacea*, EE—*Elymus elongatus*, LP—*Lolium perenne*, MG—*Miscanthus x giganteus*, PV—*Panicum virgatum*, SP—*Spartina pectinate.* Different letters indicate the presence of statistically significant differences at *p* < 0.05 according to one-way ANOVA and Tukey test.

Figure 5 and Figure 6 show the thickness swelling and water absorption of the boards after soaking in water for 24 h. The measured values of thickness swelling (TS) and water absorption (WA) of the boards in groups C_3_ and C_4_ indicate that the boards differ significantly in terms of the tested properties. Based on statistical analysis of the mean values of TS and WA for boards from the C_3_ and C_4_ groups, it was found that there were also significant differences between the groups. For particleboards with added alternative particles, the lowest TS value was observed for MG board (21.5%) and the lowest WA for PV board (72.1%). The highest values of TS (29.6%) and WA (92.1%) were recorded for FA board. The EN 312 standard does not define a maximum value of swelling in thickness after soaking in water for boards for interior fitments including furniture.

The higher thickness swelling and water absorption of boards with the addition of alternative lignocellulosic raw materials may be caused by the presence of parenchyma cells in the pith of these plants. The high hygroscopicity of the parenchyma and its spongy structure compared with other cells means that it has high capacity for water absorption and storage in the tissue cell [50].

### 3.3. Density of Particleboards

**Figure 7:**
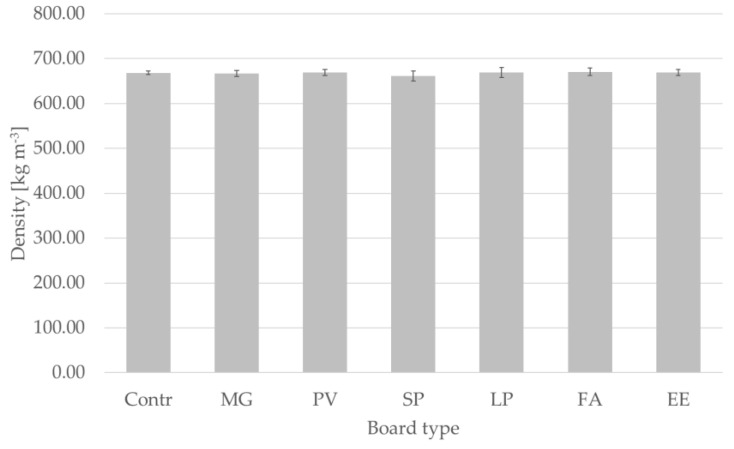
Density of manufactured panels. Abbreviations used: contr.—control (100% wood); FA—*Festuca arundinacea*, EE—*Elymus elongatus*, LP—*Lolium perenne*, MG—*Miscanthus x giganteus*, PV—*Panicum virgatum*, SP—*Spartina pectinate.* No statistically significant difference among the various boards was detected by one-way ANOVA.

No significant differences in mean density were found between the six tested species or between the two groups of species (C_3_ vs. C_4_) (Figure 7). This suggests that the applied pressure in the manufacturing process is a more important influencing factor for particleboard density than the feedstock itself, confirming the findings of Klímek et al. (2018) who reported no difference of density in particleboards produced with miscanthus and spruce [35]. However, this is true when the grinded particles have similar particle size distribution; indeed, coarser particles imply lower density of the obtained panel [55]. The average density of the tested particleboards was 667.8 ± 8.8 kg·m^−3^, while for the control board it was 668.5 ± 4.2 kg·m^−3^.

**Figure 8:**
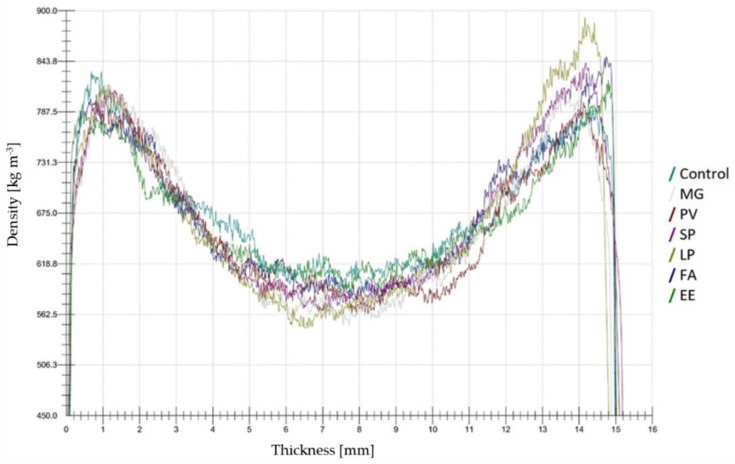
Density profiles of tested boards. FA—*Festuca arundinacea*, EE—*Elymus elongatus*, LP—*Lolium perenne*, MG—*Miscanthus x giganteus*, PV—*Panicum virgatum*, SP—*Spartina pectinate*.

The density profiles of the tested boards (Figure 8) were found to be nearly symmetrical on both sides along the central board thickness, which is typical in the case of such experiments [21,56,57]. The density was very low at the surface of each board but increased to reach the highest density at 1–2 mm from the surface. It then reduced to a constant value along the core layer and reached the next peak close to the bottom of the board. The higher density in the surface layers results in correspondingly higher bending strength, closer and more even surfaces for veneering, laminating, or painting, higher resistance to absorption and swelling, and higher resistance to ignition and the spread of flame [21].

### 3.4. Formaldehyde Content

**Figure 9:**
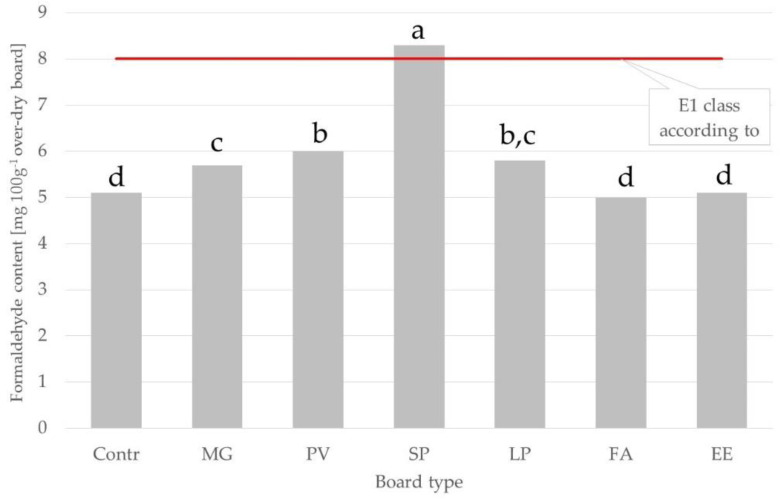
Formaldehyde content in tested particle boards made with the addition of biomass from different grass species. Abbreviations used: contr.—control (100% wood); FA—*Festuca arundinacea*, EE—*Elymus elongatus*, LP—*Lolium perenne*, MG—*Miscanthus x giganteus*, PV—*Panicum virgatum*, SP—*Spartina pectinate.* Different letters indicate the presence of statistically significant differences at *p* < 0.05 according to one-way ANOVA and Tukey test.

Mean values of formaldehyde content were significantly different between the tested grass species and between the groups of species (Figure 9). The lowest values were found for boards made with biomass from tall fescue and tall wheatgrass (5.0 and 5.1 mg·100g^−1^ oven-dry board, respectively), being comparable to the value for the control board (5.1 mg·100g^−1^ over-dry board). The highest concentrations of formaldehyde were obtained for cordgrass and switchgrass (8.3 and 6.0 mg·100g^−1^ over-dry board, respectively). The mean value for C_4_ grass species (6.7 mg·100g^−1^ over-dry board) was significantly higher than the value for C_3_ grass species (5.3 mg·100g^−1^ over-dry board). It is worth highlighting that only the values for cordgrass were higher than the threshold for the E1 class according to EN 312.

The formaldehyde issue should be further investigated in future research analyzing more samples, considering that there is no sufficient literature data of formaldehyde content of particleboards produced starting from biomass of perennial grass species available.

There are multiple factors that can affect the formaldehyde content of particleboards. Among these, it is possible to list pressing parameters, type and amount of the hardener, feedstock, type of resin, and molar ratio (of urea and formaldehyde), as well as the content of free formaldehyde in the resin [58]. However, in the presented study, the process parameters were fixed; therefore it is possible to suggest that the shown differences in formaldehyde content are related to the intrinsic characteristics of the various investigated lignocellulosic biomasses.

### 3.5. Future Research Directions

The presented results confirmed the hypothesis that perennial grasses biomass can be a relevant feedstock for particleboards production. However, some drawbacks are still evident. Poorer wettability and reduced mechanical properties in comparison to control panel were highlighted, confirming what is reported in the current literature [59]. One of the main challenges for the application of alternative lignocellulosic biomass in particleboards manufacturing is the fact that these alternative raw materials including grasses, straw, etc. are generally characterized by lower bulk density in comparison to wood raw material [60,61]. Lower bulk density of alternative raw materials, especially in the case of increasing share of them in the board, can cause some technical limitations in the manufacturing process at the industrial scale. Moreover, some logistic issues in terms of developing optimal and sustainable transport mechanism could appear.

Therefore, the future directions related to application of alternative lignocellulosic raw materials including perennial grasses to the production of particleboards should be focused on optimization of manufacturing parameters taking into account technical possibilities of the wood-based panels companies.

Furthermore, agronomic studies are needed to further test and implement the possibility of cultivating such species in marginal conditions, thus avoiding the competition with food and feed crops.

## 4. Conclusions

In the framework of looking for alternative feedstocks for particleboards’ production, the present study aimed to substitute 30% of wood particles with six perennial grass biomasses in particleboard production.

The following species: miscanthus, switch prairie, and cordgrass, being warm-season grasses with the C_4_ photosynthetic pathway; and tall wheatgrass, tall fescue, and perennial ryegrass, being cool-season grasses with the C_3_ photosynthetic pathway, were tested.

This research represents the first assessment of comparison between the quality of the particleboards of perennial grass species of different photosynthetic pathways.

As demonstrated, it is possible to state that C_4_ grasses particleboards achieved sufficient mechanical properties, even if the formaldehyde content was higher than that of the control panel. On the other hand, particleboards made with C_3_ grasses exhibited not as high mechanical properties, but demonstrated lower formaldehyde content. The obtained results demonstrated that it is possible to obtain particleboards partially based on perennial grasses biomass that meet the standard requirements for boards’ quality.

Future studies should be addressed to improve the overall quality of particleboards produced with perennial grasses biomass, including the application of alternative adhesives or pre-treatments, as well as to optimize the manufacturing parameters for the production of particleboards from perennial grasses biomass. Moreover, further agronomic studies regarding the cultivation of these grass species are needed to enable successful growing in marginal land conditions, in order to avoid competition with food crops, which is currently the main obstacle to the cultivation of many industrial crops.

## Figures and Tables

**Table 1:** Details of the establishment of field plots with perennial grasses for biomass collection.

Species Name	Cultivar Name/Breeder	Plots Established from:	Plants or Seed per m^2^	Distance in Rows/between Rows (m)
C_4_ species:				
Miscanthus (MG)	unknown	rhizomes	1	1/1
Switchgrass (SG)	MARDAN/PBAI *, POL	seedlings	2	
Cordgrass (SP)	unknown	rhizomes	2	
C_3_ species:				
Tall wheatgrass (EE)	BAMAR/BS ** Bartążek, POL	seed	10 g	0.5/0.3
Tall fescue (FA)	BAROLEX/BERENBRUG, NLD		10 g	0.5/0.3
Perennial ryegrass (LP)	BARONKA/BS Bartążek, POL		20 g	0.3/0.3

* PBAI—Plant Breeding and Acclimatization Institute, ** BS—Breeding Station.

**Table 2:** Parameters of particleboard production.

Parameter	Value/Units
Board density	670 kg·m^−3^
Board thickness	16 mm
Resination ratio	10%
Paraffin emulsion content	0.3%
Press temperature	200 °C
Pressing pressure	20.5 MPa
Pressing time	8 s·mm^−1^
Hardener ratio	3%
Hardener	40% solution of NH_4_NO_3_

**Table 3:** Properties of the adhesive.

Properties	MUF
Solid	68.3%
Viscosity	257 mPa·s
pH	8.6
Gel point (100 °C) *	95 s

* with 3% hardener added.

**Table 4:** Raw materials used in board production.

Board Type	Lignocellulosic Raw Material (Percentage Share)
Control	Wood particles (100%)
C_4_ group	
MG	Wood particles (70%), miscanthus particles (30%)
PV	Wood particles (70%), switchgrass particles (30%)
SP	Wood particles (70%), cordgrass particles (30%)
C_3_ group	
LP	Wood particles (70%), perennial ryegrass particles (30%)
FA	Wood particles (70%), tall fescue particles (30%)
EE	Wood particles (70%), tall wheatgrass particles (30%)

## Data Availability

Data available on request from the corresponding author.
